# Sterol regulatory element‐binding protein‐1c orchestrates metabolic remodeling of white adipose tissue by caloric restriction

**DOI:** 10.1111/acel.12576

**Published:** 2017-03-03

**Authors:** Namiki Fujii, Takumi Narita, Naoyuki Okita, Masaki Kobayashi, Yurika Furuta, Yoshikazu Chujo, Masahiro Sakai, Atsushi Yamada, Kanae Takeda, Tomokazu Konishi, Yuka Sudo, Isao Shimokawa, Yoshikazu Higami

**Affiliations:** ^1^ Laboratory of Molecular Pathology and Metabolic DiseaseFaculty of Pharmaceutical SciencesTokyo University of Science2641 YamazakiNoda, Chiba278‐8510Japan; ^2^ Translational Research Center, Research Institute of Science and TechnologyTokyo University of Science2641 YamazakiNoda, Chiba278‐8510Japan; ^3^ Department of Internal Medicine ResearchSasaki InstituteSasaki Foundation2‐2 KandasurugadaiChiyoda‐ku, Tokyo101‐0062Japan; ^4^ Faculty of Bioresource SciencesAkita Prefectural UniversityShimoshinjoNakano, Akita010‐0195Japan; ^5^ Department of PathologyNagasaki University Graduate School of Biomedical Sciences1‐12‐4 SakamotoNagasaki852‐8523Japan

**Keywords:** caloric restriction (CR), white adipose tissue (WAT), mitochondria biogenesis, oxidative stress, sterol regulatory element binding protein‐1c (Srebp‐1c), peroxisome proliferator‐activated receptor gamma coactivator‐1α (Pgc‐1α)

## Abstract

Caloric restriction (CR) can delay onset of several age‐related pathophysiologies and extend lifespan in various species, including rodents. CR also induces metabolic remodeling involved in activation of lipid metabolism, enhancement of mitochondrial biogenesis, and reduction of oxidative stress in white adipose tissue (WAT). In studies using genetically modified mice with extended lifespans, WAT characteristics influenced mammalian lifespans. However, molecular mechanisms underlying CR‐associated metabolic remodeling of WAT remain unclear. Sterol regulatory element‐binding protein‐1c (Srebp‐1c), a master transcription factor of fatty acid (FA) biosynthesis, is responsible for the pathogenesis of fatty liver (steatosis). Our study showed that, under CR conditions, Srebp‐1c enhanced mitochondrial biogenesis via increased expression of peroxisome proliferator‐activated receptor gamma coactivator‐1α (Pgc‐1α) and upregulated expression of proteins involved in FA biosynthesis within WAT. However, via Srebp‐1c, most of these CR‐associated metabolic alterations were not observed in other tissues, including the liver. Moreover, our data indicated that Srebp‐1c may be an important factor both for CR‐associated suppression of oxidative stress, through increased synthesis of glutathione in WAT, and for the prolongevity action of CR. Our results strongly suggested that Srebp‐1c, the primary FA biosynthesis‐promoting transcriptional factor implicated in fatty liver disease, is also the food shortage‐responsive factor in WAT. This indicated that Srebp‐1c is a key regulator of metabolic remodeling leading to the beneficial effects of CR.

## Introduction

Caloric restriction (CR) is the most robust, reproducible, and simple experimental manipulation known to extend lifespan and delay onset of many age‐associated pathophysiological changes in various laboratory rodents. Suppression of growth hormone/insulin‐like growth factor (GH/IGF‐1) signaling, reduction of mTOR signaling, activation of sirtuin, enhanced mitochondrial biogenesis, reduced oxidative stress, and suppressed inflammation mediate many of the beneficial effects of CR. However, the exact underlying mechanisms are still being debated (Chung *et al*., 2013; Guarente, 2013).

Fat‐specific insulin receptor knockout (FIRKO) mice lived longer than their controls (Blüher *et al*., 2003). Transcription factors, including CCAAT/enhancer‐binding protein (C/EBP)‐α, C/EBPβ, and peroxisome proliferator‐activated receptor‐γ (PPARγ), are master regulators of adipocyte differentiation (Farmer, 2006). Mice in which C/EBPα was replaced with C/EBPβ (β/β mice) lived longer than their wild‐type (WT) counterparts (Chiu *et al*., 2004). In contrast, hetero‐deficient PPARγ knockout (KO) mice exhibited a shortened lifespan (Argmann *et al*., 2009). White adipose tissue (WAT) is a primary harbor of inflammatory cells within obese and aged individuals, while WAT inflammation contributes to systemic metabolic dysfunction including insulin resistance and cardiovascular disease (Lumeng *et al*., 2011; Ouchi *et al*., 2011). Thus, the characteristics of WAT seem to influence age‐associated pathophysiology and the lifespan of rodents.

Sterol regulatory element‐binding proteins (SREBPs) are master transcriptional regulators of lipid metabolism with three known isoforms: SREBP‐1a, SREBP‐1c, and SREBP‐2. Both SREBP‐1 isoforms activate transcription of genes involved in fatty acid (FA) biosynthesis. SREBP‐1c is the primary isoform expressed in insulin‐sensitive tissues such as the liver, WAT, and muscle (Shimano, 2009). Because CR further extended lifespan of two long‐lived strains, Ames dwarf mice and heterozygous antisense GH transgenic rats (Bartke *et al*., 2001; Shimokawa *et al*., 2003), we examined the role of GH/IGF‐1 in CR‐associated gene expression profiles of WAT in a previous study. Our findings suggested that CR‐associated alterations of gene expression were highly regulated, in a GH/IGF‐1‐independent manner. In particular, CR downregulated inflammatory gene expression in a GH/IGF‐1‐independent manner (Chujo *et al*., 2013). In contrast, CR upregulated expression of Srebp‐1c (the mouse homolog of human SREBP‐1c) and its downstream target genes, especially those involved in FA biosynthesis, also in a GH/IGF‐1‐independent manner (Okita *et al*., 2012; Chujo *et al*., 2013). Moreover, CR significantly increased *de novo* FA biosynthesis in WAT but not in the liver (Bruss *et al*., 2010). Therefore, we hypothesized that activation of *de novo* FA biosynthesis via Srebp‐1c may be pivotal in CR‐associated metabolic remodeling of WAT, systemic metabolism and that of various organs, and longevity. To test our hypothesis, CR‐associated responses in Srebp‐1c KO and their embryonic fibroblasts (MEFs) were compared with those of wild‐type mice (WT).

## Results

### Srebp‐1c was required for CR‐associated activation of fatty acid biosynthesis in WAT

Food intake was significantly higher in KO than in WT until almost 20 months of age. At 8–10 months of age, body weight was also higher in KO than in WT and the effects of CR on body weight were slightly attenuated in KO. The effects of CR on the weights of the tissues examined in our study were not significantly different in WT and KO (Fig. S1; Table S1, Supporting information). Plasma levels of nonesterified fatty acids and 3‐hydroxybutyric acid in *ad libitum* (AL)‐fed KO (KOAL) were markedly lower than in fasted WT. Moreover, KO had a higher respiratory quotient and were more vulnerable to starvation than the WT (unpublished data). These data suggested that KO may not adjust to food shortage as compared with WT. Plasma insulin was significantly lower under fasted conditions for all four groups of mice. Plasma levels of IGF‐1 and leptin were significantly lower in CR than in AL, particularly under fed and fasted conditions, respectively. However, the effects of CR did not differ in WT and KO. In KO, plasma adiponectin levels were markedly increased under fasted, compared with fed conditions, but this fasting‐associated phenotype was not found in WT. In addition, plasma levels of insulin, IGF‐1, adiponectin, and leptin were slightly elevated in KO (Table 1).

**Table 1:** Plasma parameter

Plasma parameter	WT				KO			
	AL		CR		AL		CR	
	Fed	Fasted	Fed	Fasted	Fed	Fasted	Fed	Fasted
Glucose (mg dL^−1^)	203.7 ± 9.6	110.8 ± 18.1$	176.8 ± 4.5	169.9 ± 14.2	243.7 ± 9.7	157.3 ± 21.1$	190.2 ± 9.4	200.1 ± 11.4
t‐Cho (mg dL^−1^)	72.63 ± 2.37	71.12 ± 1.65	49.90 ± 0.80*	53.31 ± 5.08	55.96 ± 0.76	62.40 ± 0.00	24.89 ± 4.24*,†	43.07 ± 2.22*,$
TG (ng dL^−1^)	107.9 ± 10.6	140.2 ± 6.5	84.1 ± 2.8	72.4 ± 4.6*	86.3 ± 14.1	103.9 ± 5.8	84.0 ± 5.9	65.9 ± 3.3
NEFA (mEq mL^−1^)	0.59 ± 0.03	1.04 ± 0.04$	0.42 ± 0.05*	0.86 ± 0.05*$	0.51 ± 0.02	0.81 ± 0.04†$	0.41 ± 0.02	0.70 ± 0.01$
3‐HB (mm)	Undetected	1.94 ± 0.15	Undetected	0.72 ± 0.05**	Undetected	0.97 ± 0.12†	Undetected	0.58 ± 0.22
Insulin (pg mL^−1^)	1575 ± 347	262 ± 92$	1373 ± 40	45 ± 4$	2234 ± 59	362 ± 43$	2158 ± 187	455 ± 95$
IGF‐1 (ng mL^−1^)	426.28 ± 11.33	322.24 ± 40.45	313.63 ± 11.59*	244.11 ± 32.21	482.20 ± 18.75	360.20 ± 20.40$	347.02 ± 29.11**	306.41 ± 8.42
Adiponectin (μg mL^−1^)	8.97 ± 0.41	8.91 ± 0.67	10.87 ± 0.32	11.70 ± 0.37	9.60 ± 0.91	14.17 ± 1.43†$	13.03 ± 1.14	11.61 ± 1.04
Leptin (ng mL^−1^)	12.80 ± 2.09	8.98 ± 1.86	5.82 ± 0.73	1.07 ± 0.21***,$$$	25.92 ± 6.03	17.66 ± 6.38	8.50 ± 1.33*	3.40 ± 0.45**,†

t‐Cho: total cholesterol, TG: triglyceride, NEFA: non‐esterified fatty acid, 3‐HB: 3‐hydroxybutyric acid.

Each value represents the means ± SEM of 3–5 mice. *: *P* < 0.05 vs. AL, †: *P* < 0.05 vs. Wild, $: *P* < 0.05 vs. fed analyzed by Tukey's test.

**Figure 1:**
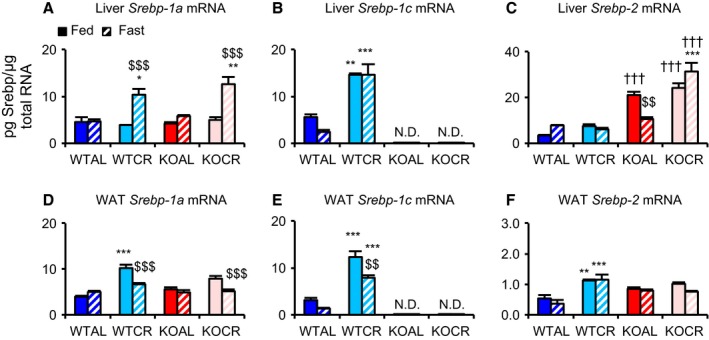
Effects of CR on *Srebp* expression in the liver and white adipose tissue (WAT). (A–C) Copy numbers of *Srebp‐1a* (A), *Srebp‐1c* (B), and *Srebp‐2* (C) mRNAs in liver tissue from wild‐type mice (WT) and knockout mice (KO) fed *ad libitum* (AL) and subjected to 30% caloric restriction (CR). (D–F) Copy numbers of *Srebp‐1a* (D), *Srebp‐1c* (E), and *Srebp‐2* (F) mRNAs in WAT samples from WT and KO mice fed AL and subjected to 30% CR. Four groups of mice were further divided and given two treatments (fed or fasted). Accordingly, eight groups of mice (WTAL‐fed, WTAL‐fast, WTCR‐fed, WTCR‐fast, KOAL‐fed, KOAL‐fast, KOCR‐fed, KOCR‐fast) were euthanized at 8–10 months of age (*n* = 3–6 per group). Values shown in all panels are means ± SEM. *P <* *0.05, **P <* *0.01, ***P <* *0.001 vs. AL, †††*P *<* *0.001 vs. WT, $$*P *<* *0.01, $$$*P *<* *0.001 vs. fed, analyzed by Tukey's test.

**Figure 2:**
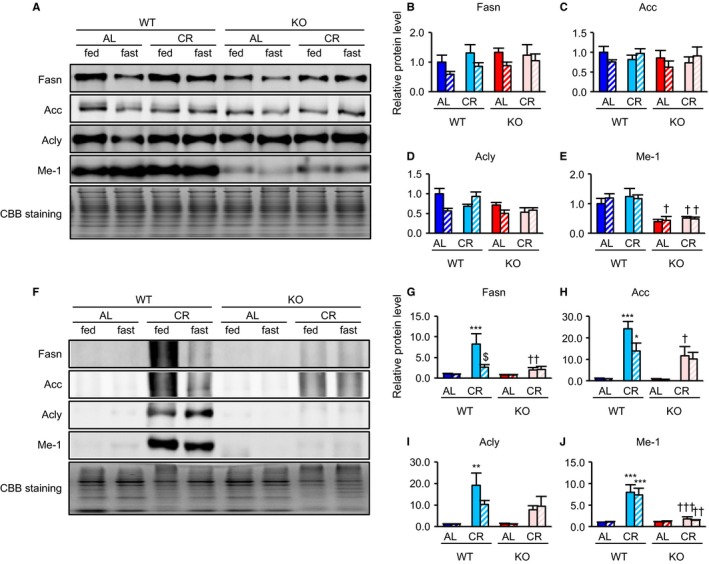
Srebp‐1c was required for CR‐associated upregulation of proteins involved in fatty acid (FA) biosynthesis in WAT but not in the liver. (A–E) Liver and (F–J) WAT. Example of immunoblot images showing expression of proteins involved in FA biosynthesis in the liver (A) and WAT (F) from eight groups of mice (WTAL‐fed, WTAL‐fast, WTCR‐fed, WTCR‐fast, KOAL‐fed, KOAL‐fast, KOCR‐fed, KOCR‐fast). Quantitative analysis of immunoblots was performed using a chemiluminescence method. Results for levels of Fasn (B, G), Acc (C, H), Acly (D, I), and Me‐1 (E, J) are expressed as relative intensity of indicated protein/Coomassie brilliant blue (CBB) staining compared with in the WTAL‐fed group (n = 4 per group). Values shown in all panels are means ± SEM. *P <* *0.05, **P <* *0.01, ***P <* *0.001 vs. AL, †*P *<* *0.05, ††*P *<* *0.01 and †††*P *<* *0.001 vs. WT, $*P *<* *0.05 vs. fed, analyzed by Tukey's test.

The effects of CR on *Srebp* expression in the liver and WAT were analyzed by assessing mRNA copy numbers present for each isoform. In liver tissue, CR increased *Srebp‐1a* mRNA expression only in fasted WT and KO. By comparison, CR enhanced expression of *Srebp‐1c* in both fed and fasted WT. In contrast, *Srebp‐2* mRNA expression was upregulated in KO, particularly in CR animals (Fig. 1A–C). In WAT, CR significantly increased *Srebp‐1a* mRNA expression in WT, but not in KO, when fed. CR also markedly enhanced *Srebp‐1c* mRNA expression in WT, with CR‐associated upregulation exaggerated under fed conditions (Fig. 1D,E). Moreover, CR significantly upregulated *Srebp‐2* mRNA expression in WT, but not in KO (Fig. 1F). Unexpectedly, in the liver, CR did not upregulate expression of proteins involved in FA biosynthesis under any conditions tested in this study. However, malic enzyme 1 (Me‐1) protein was markedly downregulated in both fed and fasted KO mice (Fig. 2A–E). In accordance with *Srebp‐1c* mRNA levels in WAT, CR upregulated expression of downstream targets of Srebp‐1c, including fatty acid synthase (Fasn), acetyl‐CoA carboxylase (Acc), ATP citrate lyase (Acly), and Me‐1 proteins, in both fed and fasted WT, but not in KO mice (Fig. 2F–J). In kidney and quadriceps femoris muscle (QFM), it appeared that CR slightly upregulated expression of proteins involved in FA biosynthesis in WT, although this was attenuated in KO (Fig. S2A–I, Supporting information). However, because of variability caused by individual differences in protein expression, several differences between these two mouse strains did not achieve statistical significance and, therefore, represented only trends. In cardiac tissue, CR did not upregulate expression of these proteins in either WT or KO (Fig. S2J–M, Supporting information).

### Srebp‐1c was required for CR‐associated activation of mitochondrial biogenesis in WAT, but not in other tissues

**Figure 3:**
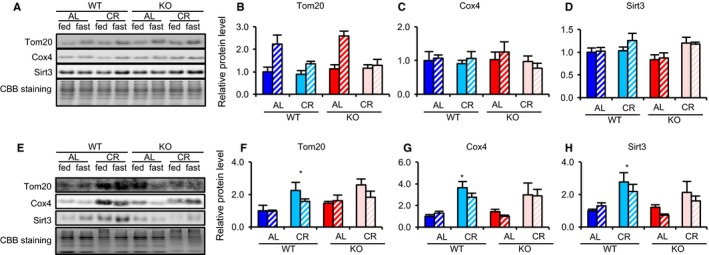
Srebp‐1c was required for CR‐associated upregulation of proteins involved in mitochondrial biogenesis in WAT but not in the liver. (A–D) Liver and (E–H) WAT. Example of immunoblot images showing expression of proteins involved in mitochondrial biogenesis in liver tissue (A) and WAT (E) samples from eight groups of mice (WTAL‐fed, WTAL‐fast, WTCR‐fed, WTCR‐fast, KOAL‐fed, KOAL‐fast, KOCR‐fed, KOCR‐fast). Quantitative analysis of immunoblots was performed using a chemiluminescence method. Results for Tom20 (B, F), Cox4 (C, G), and Sirt3 (D, H) are each expressed as relative intensity of the indicated protein/CBB staining compared with values in the WTAL‐fed group (*n* = 4 per group). Values in all panels are means ± SEM. *P <* *0.05 vs. AL, analyzed by Tukey's test.

**Figure 4:**
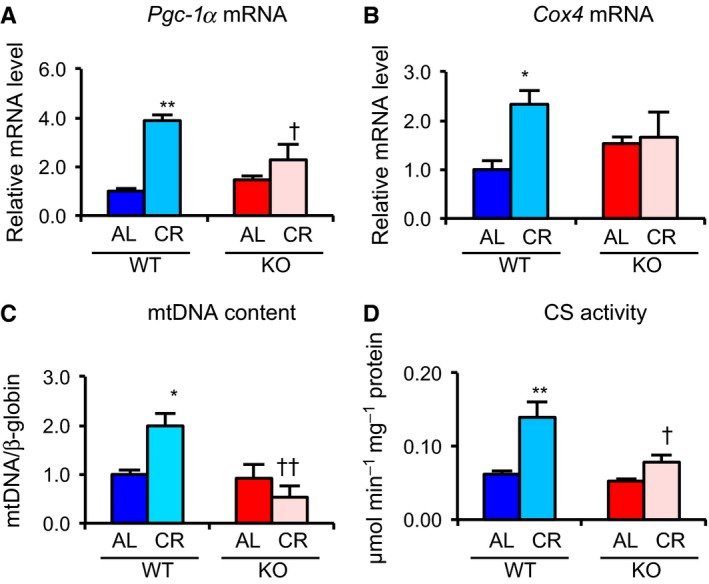
Srebp‐1c was required for CR‐associated activation of mitochondrial biogenesis in WAT. (A, B) mRNA expression levels of mitochondrial‐related genes, *Pgc‐1α* (A) and *Cox4* (B), in WAT from four groups of fed mice were analyzed by real‐time RT–PCR. Data were normalized against values for *Tbp* expression (*n* = 3–5). (C) Ratio of mitochondrial (COX2) vs. nuclear (β‐globin) DNA in WAT from four groups of fed mice was obtained by real‐time PCR (*n* = 4–6). (D) Citrate synthase (CS) activity in WAT from four groups of fed mice was measured spectrophotometrically at 412 nm. Values in all panels are means ± SEM. *P <* *0.05, **P <* *0.01 vs. AL, †*P *<* *0.05 and ††*P *<* *0.01 vs. WT, analyzed by Tukey's test.

In various reports, CR enhanced mitochondrial biogenesis in several tissues (Nisoli *et al*., 2005; Finck & Kelly, 2006). Two long‐lived strains, FIRKO and β/β mice, showed, compared with WT mice, decreased adiposity and enhanced mitochondrial biogenesis in WAT (Chiu *et al*., 2004; Katic *et al*., 2007). To clarify the impact of Srebp‐1c on CR‐enhanced mitochondrial biogenesis, we analyzed three mitochondrial proteins, translocase of outer mitochondrial membranes 20 kDa (Tom20), cytochrome c oxidase subunit 4 (Cox4), and sirtuin 3 (Sirt3). CR did not increase expression of these proteins in the liver, kidney, QFM or heart from either WT or KO (Fig. 3A–D and S3, Supporting information). CR also did not increase citrate synthase (CS) activity in those tissues from either strain (Fig. S4, Supporting information). In contrast, in WAT, CR enhanced expression of these proteins, but this effect was lower in KO than in WT (Fig. 3E–H). PPARγ coactivator‐1α (Pgc‐1α) plays a critical role in CR‐associated mitochondrial biogenesis (Anderson *et al*., 2008). In WAT, CR significantly upregulated expression of *Pgc‐1α* and *Cox4* mRNAs in WT, but not in KO (Fig. 4A,B). Similarly, CR increased mitochondrial DNA (mtDNA) content and CS activity in WT, but not in KO (Fig. 4C,D). This suggested that CR enhanced mitochondrial biogenesis via Srebp‐1c in WAT, but not in other tissues.

### Srebp‐1c enhanced mitochondrial biogenesis via transcriptional activation of *Pgc‐1a*

**Figure 5:**
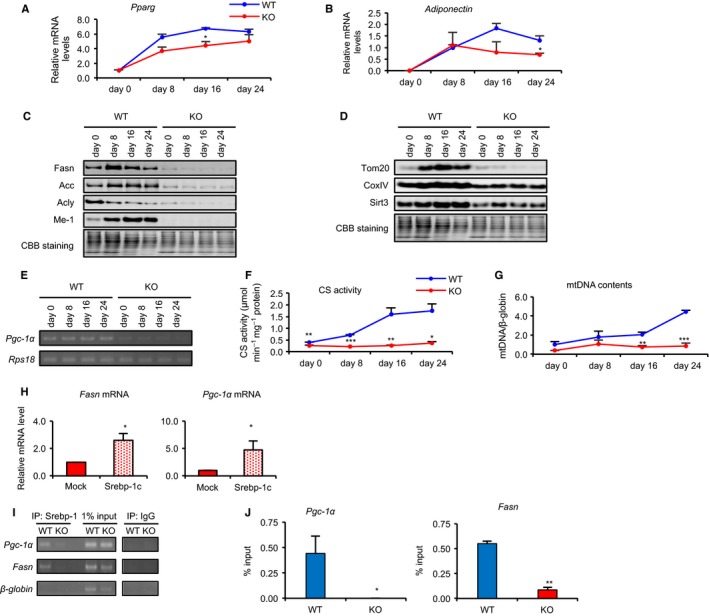
Srebp‐1c enhanced mitochondrial biogenesis via transcriptional activation of *Pgc‐1a*. (A, B) mRNA expression levels of adipocyte differentiation‐related genes, *Pparg* (A) and *adiponectin* (B), in WAT from four groups of fed mice were analyzed by real‐time RT–PCR. Data were normalized against values for *Tbp* expression (*n* = 3). (C, D) Example of immunoblot images showing expression of proteins involved in FA biosynthesis (C) and mitochondria (D) during adipocyte differentiation in primary MEFs derived from WT and KO mice, respectively. (E) Representative gel image of RT–PCR showing expression of *Pgc‐1α* genes in primary MEFs. (F) CS activity in MEFs from WT and KO was measured spectrophotometrically at 412 nm. (G) Ratio of mitochondrial (COX2) vs. nuclear (β‐globin) DNA in MEFs from WT and KO was obtained by real‐time PCR (*n* = 3). (H) Transfection of the mature form of Srebp‐1c expression vector in KO‐MEFs. The mRNA expression levels of *Fasn* and *Pgc‐1α* were analyzed by real‐time RT–PCR. Data were normalized to values for *Rps‐18* expression. Values are means ± SEM (*n* = 3). *P <* *0.05 by Student's *t*‐test. (I) SREBP‐1c is occupied on *Pgc‐1* and *Fasn* promoters in MEFs. Representative gel image of ChIP assay for Srebp‐1c enrichment on *Pgc‐1α* and *Fasn* promoters. PCRs for promoter of *Pgc‐1α*,* β‐globin*, and *Fasn* genes were performed using DNA pulled down with Srebp‐1 antibody–transcription factor complexes in WT‐ and KO‐MEFs. (J) ChIP assay was performed by real‐time PCR, using the same DNA–Srebp‐1 antibody–transcription factor complex samples as for (I). Percent (%) input was calculated using the formula: 2^(Ct [1% of input]−Ct [ChIP])^. For ChIP assays, IgG was used as a negative control. Experiments were each run twice, with similar results.

To further clarify the role of Srebp‐1c in CR‐associated metabolic alterations within WAT, we examined protein expression in MEFs during adipocyte differentiation, comparing those derived from KO (KO‐MEFs) and WT (WT‐MEFs). First, to confirm whether MEFs of both genotypes were equivalently differentiated to mature adipocytes, we analyzed mRNA expression of two adipocyte differentiation markers, *Pparg* and *adiponectin*. Expression of both genes was upregulated almost equally in KO‐ and WT‐MEFs (Fig. 5A,B). However, expression of proteins involved in FA biosynthesis and mitochondrial biogenesis was significantly higher in WT‐MEFs than in KO‐MEFs (Fig. 5C,D). Furthermore, expression of *Pgc‐1α* mRNA was markedly higher in WT‐MEFs (Fig. 5E). Similarly, elevation of both CS activity and mtDNA content was observed in WT‐MEFs, but not in KO‐MEFs, during adipocyte differentiation (Fig. 5F,G). These findings suggested that decreased levels of proteins involved in FA biosynthesis and mitochondrial biogenesis in KO‐MEFs resulted from deletion of Srebp‐1c, rather than from inhibition of adipogenesis.

To clarify effects of Srebp‐1c on *Pgc‐1α* transcription, we examined whether Srebp‐1c overexpression in Srebp‐1c KO‐MEFs would rescue *Pgc‐1α* mRNA expression levels. Indeed, overexpression of the mature form of SREBP‐1c rescued *Pgc‐1α* mRNA expression, as well as the level of *Fasn* mRNA (Fig. 5H). Because two sterol regulatory elements are believed to bind to Srebp‐1c, between −500 bp and 0 bp of the *Pgc‐1α* promoter, we examined whether CR increased Srebp‐1 binding in the *Pgc‐1α* promoter region in WAT from rats. We used rats subjected to CR for this experiment because sufficient quantities of WAT could not be obtained from mice. Results from a chromatin immunoprecipitation (ChIP) assay incorporating an antibody against Srebp‐1 suggested that more binding occurred in CR than in AL rats (Fig. S5, Supporting information). As no specific antibodies against Srebp‐1a or Srebp‐1c are currently available, we used ChIP to determine whether Srebp‐1a or Srebp‐1c could occupy the *Pgc‐1α* promoter region in WT‐ and KO‐MEFs. These results showed that SREBP‐1c could directly activate transcription of *Pgc‐1a* and *Fasn* (Fig. 5I,J).

### Srebp‐1c was required for CR‐associated suppression of oxidative stress in WAT and for prolongevity effects

**Figure 6:**
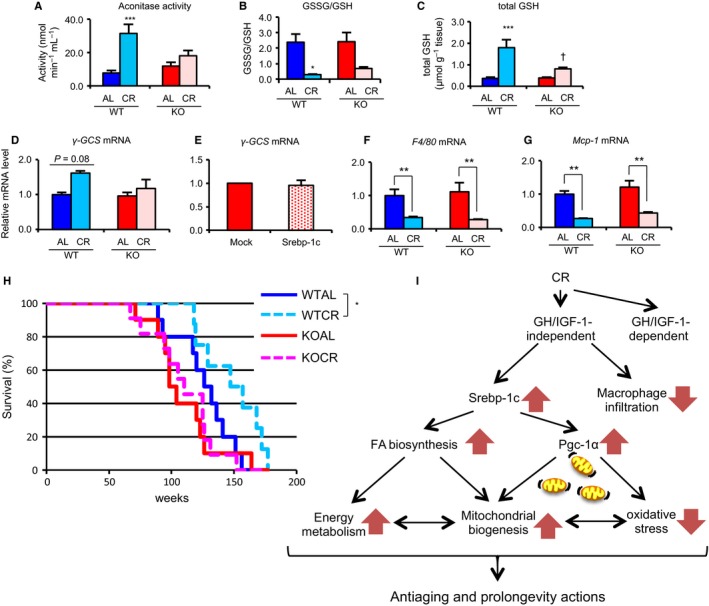
Srebp‐1c was required for CR‐associated suppression of oxidative stress and prolongevity effects but was not required for macrophage infiltration in WAT. (A–D) Various biomarkers for oxidative stress, including aconitase activity (A), glutathione disulfide (GSSG)‐to‐glutathione (GSH) ratio (B), total GSH (C), and expression level of *γ‐Gcs* mRNA (D), were measured in WAT from four groups of fed mice (*n* = 3–5 per group). (A) Aconitase activity was spectrophotometrically determined at 340 nm monitoring production of NADPH, generated by a coupled reaction of aconitase with isocitric dehydrogenase. (B, C) GSSG‐to‐GSH ratio (B) and total GSH (C) were measured spectrophotometrically at 412 nm by a 5,5′‐dithio‐bis(2‐nitrobenzoic acid)‐GSSG reductase (DTNB‐GSSG reductase) recycling assay. (D, F, G) mRNA expression levels of *γ‐Gcs* (D), *F4/80* (F), and *Mcp‐1* (G) in WAT from four groups of fed mice were analyzed by real‐time RT–PCR. Data were normalized to values for *Tbp* expression (*n* = 3–5). (E) Transfection of the mature form of Srebp‐1c expression vector in KO‐MEFs. The mRNA expression level of *γ‐Gcs* was analyzed by real‐time RT–PCR. Data were normalized to that for *Rps‐18* expression. Values are means ± SEM (*n* = 3). Values in all panels are means ± SEM. **P <* *0.01, ***P <* *0.001 vs. AL, †*P *<* *0.05 vs. WT, analyzed by Tukey's test. (H) Survival curve. A total of 8–11 mice in four groups were maintained under pathogen‐free condition. To compare survival distributions of AL and CR mice, the log‐rank test was applied. *P* values < 0.05 were considered statistically significant. (I) Diagram demonstrating the proposed novel molecular mechanism of CR‐associated beneficial metabolic remodeling in WAT and prolongevity effects.

As CR generally suppresses oxidative stress (Song *et al*., 2014), we evaluated two biomarkers of oxidative stress, aconitase activity and the ratio of oxidized glutathione to reduced glutathione (GSSG/GSH). In WAT, CR significantly increased aconitase activity and decreased the GSSG/GSH ratio in WT, but not in KO (Fig. 6A,B). Moreover, CR increased total GSH in WT, but had no effect on these levels in KO (Fig. 6C). γ‐Glutamylcysteine synthetase (γ‐Gcs), a rate‐limiting enzyme for GSH biosynthesis (Meister, 1991), was slightly upregulated by CR in WT, but not in KO (Fig. 6D). Collectively, these findings suggested that CR suppressed oxidative stress through regulation of GSH biosynthesis, which was mediated by *γ‐Gcs* expression in a Srebp‐1c‐dependent manner. However, overexpression of the mature form of SREBP‐1c did not increase *γ‐Gcs* mRNA expression in KO‐MEFs (Fig. 6E), suggesting that SREBP‐1c alone was insufficient to upregulate *γ‐Gcs* transcription. In liver tissue, CR did not significantly alter aconitase activity, but it significantly reduced thiobarbituric acid‐reactive substances (TBARS) in both WT and KO (Fig. S6A,C, Supporting information). This suggested that CR suppressed oxidative stress in a Srebp‐1c‐independent manner in the liver. In the kidney, QFM, and heart, CR did not significantly improve either GSSG/GSH or TBARS (Fig. S6B, C, Supporting information). Thus, Srebp‐1c was specifically required for CR‐associated suppression of oxidative stress in WAT, but not in the other tissues. Moreover, we examined the effects of Srebp‐1c on CR‐associated changes in macrophage infiltration. Within WT and KO, CR markedly and equivalently downregulated expression of macrophage markers, *F4/80* and the proinflammatory cytokine *monocyte chemoattractant protein‐1 (Mcp‐1)* (Fig. 6F,G).

Finally, we analyzed distribution of the lifespan in the four groups. They showed a linear relationship with a normal distribution (Fig. S7, Supporting information). The slopes of these regression lines were almost same among the four groups, whereas the order of their Y‐intercept values from large to small was as follows: WTCR > WTAL > KOAL = KOCR. These results indicated that both CR and KO probably altered longevity, although it could not be denied that the power of this test was insufficient to explain the effects on lifespan between WT and KO mice because of the extremely small number of mice examined in this study. However, it was clear that CR predominantly affected WT (*P* = 0.029, by the log‐rank test), because the differences among KO groups (*P* = 0.464) were negligible (Fig. 6H).

## Discussion

Previous reports demonstrated that expression of Srebp‐1c and its downstream target genes is upregulated and downregulated under fed and fasting conditions, respectively, in both the liver and WAT (Horton *et al*., 1998; Sekiya *et al*., 2007). However, we observed that CR enhanced expression of proteins involved in FA biosynthesis, via Srebp‐1c, in WAT, regardless of feeding condition. Moreover, CR upregulated expression of Srebp‐1c in the liver under both fed and fasting conditions, but did not alter expression of FA biosynthesis proteins. Thus, CR‐associated changes in FA biosynthesis protein expression differed from that of Srebp‐1c in the liver. Further experiments will be required to clarify these discrepancies and the differential responses to CR between liver and WAT.

It was previously reported that the antiaging and prolongevity actions of CR were attenuated in long‐lived GH receptor (GHR) KO mice (Bonkowski *et al*., 2006). Visceral fat removal elevated the respiratory quotient in GHRKO mice, but had the opposite effect in WT mice (Masternak *et al*., 2012). These observations suggested that WAT derived from long‐lived strains of mice, that is, good‐quality WAT, can increase whole‐body lipid utilization. Recently, we proposed that Srebp‐1c‐induced activation of FA biosynthesis is one of the major mechanisms by which CR can remodel metabolism in WAT (Okita *et al*., 2012; Chujo *et al*., 2013). Previous studies clearly demonstrated that CR significantly enhanced whole‐body lipid utilization and increased endogenously synthesized FAs in WAT, but not in the liver (Bruss *et al*., 2010). Therefore, we hypothesize that CR can induce, via Srebp‐1c, a shift of the substrate used for whole‐body energy from carbohydrate to lipid.

In our study, we also demonstrated that Srebp‐1c was responsible for CR‐associated activation of mitochondrial biogenesis and reduction of oxidative stress in WAT, but not in the other tissues. These findings are inconsistent with a previous report that CR enhanced mitochondrial biogenesis in various tissues, including WAT and liver (Nisoli *et al*., 2005). In contrast to that, however, certain reports suggested that CR did not induce mitochondrial biogenesis or increase mitochondrial content (Hempenstall *et al*., 2012; Lanza *et al*., 2012). Although the reasons for these discrepancies are unclear, they might result from differences in experimental conditions, including onset age or duration of CR, strain backgrounds of mice, diet components, and housing environments. Furthermore, it was reported that Pgc‐1α was involved in CR‐enhanced mitochondrial biogenesis (Anderson *et al*., 2008). Our results indicated that CR‐enhanced mitochondrial biogenesis was caused by upregulation of *Srebp‐1c* expression to transcriptionally enhance *Pgc‐1α* expression in WAT. In support of this, Srebp‐1c activated the PGC‐1α promoter in brown adipocytes (Hao *et al*., 2010). Our findings supported other reports that multilocular adipocytes, which are characteristically similar to brown adipocytes, were present in WAT in CR mice (Higami *et al*., 2004). Moreover, it is likely that Srebp‐1c is required for CR‐associated upregulation of *γ‐Gcs* gene expression and Sirt3 protein expression in WAT. In contrast to *Pgc‐1α*,* γ‐Gcs* transcripts were not directly and sufficiently upregulated by Srebp‐1c alone. Sirt3 can deacetylate several mitochondrial enzymes to activate their enzymatic activities (Rardin *et al*., 2013). For example, in response to CR, Sirt3 activated superoxide dismutase 2 (Sod2) (Qiu *et al*., 2010). Moreover, Sirt3 activated isocitrate dehydrogenase 2 (Idh2), thereby increasing NADPH levels in mitochondria, leading to suppression of oxidative stress (Someya *et al*., 2010). Therefore, Srebp‐1c‐dependent upregulation of Sirt3 protein, as well as enhanced GSH biosynthesis, may be vital for CR‐associated suppression of oxidative stress in WAT. In general, increased oxidative stress in WAT can cause dysregulated production of adipokines. Additionally, increased ROS production in WAT can lead to increased oxidative stress in the blood, causing harmful events to occur in various other organs (Furukawa *et al*., 2004). Certain genetically modified animals living longer than controls were reported to exhibit activated mitochondrial biogenesis in WAT (Chiu *et al*., 2004; Katic *et al*., 2007). The relationship between oxidative stress and lifespan is still controversial. For example, Pérez *et al*. (2009) found that the mice overexpressing Sod2, which plays a major role in the detoxification of superoxide anions generated in the mitochondria, did not have extended lifespan. On the other hand, Schriner *et al*. (2005) reported that the mice overexpressing human catalase in the mitochondria, which catalyzes the decomposition of hydrogen peroxide into oxygen and water, lived longer than control mice (Schriner *et al*., 2005). These findings suggested that the inactivation of mitochondrial hydrogen peroxide played an important role in the extension of lifespan, while that of superoxide anions did not. In other words, the effect on lifespan would be different depending on the type of ROS. Therefore, we hypothesize that, particularly in WAT, CR‐associated metabolic remodeling, including enhanced lipid metabolism, mitochondrial biogenesis activation, and decreased certain types of mitochondrial oxidative stress, all regulated by Srebp‐1c, may have beneficial systemic effects, preventing age‐associated pathophysiologies and leading to lifespan extension (Fig. 6I).

It is widely accepted that adiponectin and leptin are anti‐inflammatory and proinflammatory adipokines, respectively (Ouchi *et al*., 2011). Transgenic mice overexpressing adiponectin in the liver live longer than controls (Otabe *et al*., 2007), suggesting an important role for adiponectin in promoting antiaging and longevity. In our study, plasma adiponectin levels in KO were slightly higher than in WT. Particularly under AL fasted conditions, these levels were significantly higher in KO than in WT. Our data were inconsistent with the beneficial phenotypes attributed to higher adiponectin levels in other studies (Otabe *et al*., 2007). Plasma adiponectin is composed of a more functional high molecular weight form (HMW) and a less functional low molecular weight form (LMW) (Yamauchi & Kadowaki, 2013). In KO mice, therefore, LMW, rather than HMW, might be the dominant form of adiponectin. CR decreased plasma IGF‐1 and leptin levels in both WT and KO. Moreover, CR equivalently suppressed macrophage infiltration in both WT and KO. These findings suggested that CR‐associated depression of leptin levels was involved in suppressing inflammation in a Srebp‐1c‐independent manner.

In this study, we demonstrated for the first time that Srebp‐1c orchestrated CR‐associated and GH/IGF‐1‐independent regulation of metabolic remodeling through effects on lipid metabolism, mitochondrial biogenesis, and oxidative stress in WAT. Upregulation of Srebp‐1c expression was found in the liver of obese mice (Ferré & Foufelle, 2010; Kammoun *et al*., 2009). Liver‐specific Srebp‐1c transgenic mice showed hepatic steatosis (Knebel *et al*., 2012), suggesting that Srebp‐1c was responsible for the pathogenesis of this condition. In the context of long‐term energy shortage, such as that induced by CR, Srebp‐1c may induce alteration of WAT function from an energy storage system to an energy transducer capable of transforming glucose into energy‐dense lipids. Thus, Srebp‐1c can act as both a FA biosynthesis‐responsive factor in the liver and a food shortage‐responsive factor in WAT.

## Experimental procedures

### Animals

This study was conducted in accordance with provisions of the Ethics Review Committee for Animal Experimentation at Tokyo University of Science.

Srebp‐1c^+/−^ (B6; 129S6‐*Srebf1* ^*tm1Mbr*^/J) mice (Liang *et al*., 2002) were purchased from Jackson Laboratory (Bar Harbor, ME, USA). Srebp‐1c^+/+^ mice (WT) and Srebp‐1c^−/−^ mice (KO) were obtained by mating Srebp‐1c^+/−^ mice and genotyping progeny by PCR. All mice were maintained under specific‐pathogen‐free (SPF) conditions at 23 °C with 12‐h light/dark cycles in the Laboratory Animal Center at the Faculty of Pharmaceutical Sciences, Tokyo University of Science. Animals had access to water and were fed a CR‐LPF diet (Oriental Yeast, Tokyo, Japan). From 3 months of age, WT and KO were divided into two groups: One was fed *ad libitum* (AL) and the other was calorie‐restricted (CR; 70% of AL energy intake, independently for each line). At 8–10 months of age, mice that were group‐housed were euthanized under anesthesia with isoflurane inhalation (Mylan, Canonsburg, PA, USA). Prior to euthanasia, mice in the CR and AL groups were further divided to receive two treatments (fed or fasted) as follows. WTCR‐fed and KOCR‐fed groups were provided with food 30 min before turning off the lights in the evening and were sacrificed 1–3 h later. To evaluate the effects of fasting, half of the CR mice were fasted overnight (approximately 20 h) prior to sacrifice (WTCR‐fast and KOCR‐fast). Similar to CR mice, half of the AL mice were sacrificed 20 h after removal of food from their cages, which occurred when the lights were turned off (WTAL‐fast and KOAL‐fast), while the other half were sacrificed without removing food (WTAL‐fed and KOAL‐fed). When mice were euthanized, epididymal adipose tissue (WAT), liver, kidney, quadriceps femoris muscle (QFM), and heart samples were collected and weighed, as shown in Table S1.

Male 5‐ to 7‐week‐old Wistar rats and their husbandry care and diet were as previously described (Okita *et al*., 2012), and their use is described under additional experimental procedures in Data S1 (Supporting information). When the animals were euthanized, epididymal WAT samples were collected. Tissues were immediately diced, frozen in liquid nitrogen, and stored at −80°C until analysis. Blood samples were collected in 1.5‐mL microtubes with ethylenediaminetetraacetic acid (EDTA). After centrifugation (2500 × *g*, 10 min, 4 °C), plasma samples were stored at −80 °C until analysis.

### Plasma biochemical analyses

Plasma glucose, insulin, adiponectin, and leptin levels were measured by Autokit Glucose (Wako, Osaka, Japan), Mouse Insulin ELISA KIT (U‐type) (Shibayagi, Japan), Quantikine^®^ ELISA Mouse Adiponectin/Acrp30 Immunoassay (R&D Systems, Minneapolis, MN, USA), and Quantikine^®^ ELISA Mouse/Rat Leptin Immunoassay (R&D Systems), respectively. t‐Cho, TG, and NEFA were measured with LabAssay^™^ Cholesterol (Wako), LabAssay^™^ Triglyceride (Wako), and LabAssay^™^ NEFA (Wako), respectively. All assays were performed according to the manufacturers' protocols. Plasma 3‐HB levels were measured by modification of a previously reported method (Hansen & Freier, 1978). Briefly, plasma was added to a reaction buffer, containing 80 mm Tris–HCl (pH 9.5) and 4 mm β‐NAD^+^, and incubated at 37 °C for 5 min. Reactions were initiated by addition of 0.37 U mL^−1^ 3‐hydroxybutyrate dehydrogenase. Changes in absorbance were measured for 10 min at 340 nm using a SpectraMax Plus384 (Molecular Devices, Sunnyvale, CA, USA).

### Preparation of MEFs, cell culture and adipocyte differentiation

Male and female Srebp‐1c^+/−^ mice were crossed, and MEFs were prepared from pregnant females. Each 13‐ to 15‐day‐old embryo was dissected from the uterus and washed with PBS. After removal of the head, tail, limbs, and blood‐enriched organs, the remaining tissue was washed with PBS, minced, and trypsinized at 37 °C for 10 min. After inactivation of trypsin by adding fetal bovine serum (FBS; Sigma‐Aldrich, St. Louis, MO, USA), MEFs were separated by filtration through a cell strainer. Cells were cultured and passaged in MEM High Glucose (Wako) with 10% FBS, 1% penicillin and streptomycin (Sigma), and 0.1 μM 2‐mercaptoethanol (Sigma). To induce adipocyte differentiation, MEFs were cultured until confluence. At confluence, maintenance medium was changed to MEF differentiation medium, containing 500 μM 3‐isobutyl‐1‐methylxanthine (Sigma), 1 μM dexamethasone (Sigma), 10 μg mL^−1^ insulin (Sigma), and 100 μM troglitazone (Wako). The differentiation medium was changed every other day and used for quantitative real‐time RT–PCR, Western blotting, and ChIP assay. KO‐MEFs were also infected with retrovirus expressing Srebp‐1c.

### Quantitative real‐time RT–PCR

Total RNA was extracted from frozen WAT and liver tissue and quantitative real‐time PCR (qRT–PCR) was performed as described previously (Chujo *et al*., 2013). The methods are briefly described as additional experimental procedures in Data S2 (Supporting information).

### Construction of *Srebp‐1c* expression vector

Mouse cDNA libraries were generated by reverse transcription of total RNA from liver as described above. The coding region of the mature form of the *Srebp‐1c* gene was amplified from mouse cDNA using the following primers: forward primer 5′‐GTC GAC CAC CAT GGA ACA AAA ACT CAT CTC AGA AGA GGA TCT GGA CTA CAA AGA CGA TGA CGA CAA GGG AGC CAT GGA TTG CAC ATT TGA AGA‐3′ and reverse primer 5′‐ GTC GAC TTA GTG GTG GTG GTG GTG GTG CAG GGC CAG GCG GGA G ‐3′. PCR was performed using PrimeSTAR HS polymerase (Takara, Japan) according to the manufacturer's protocol. The PCR product was ligated into a pBluescript II KS(+) digested with *Eco*RV. The sequence of the insert was confirmed by DNA sequencing using the BigDye Terminator v3.1 Cycle Sequencing Kit (Applied Biosystems, CA, USA) and a 3100 Genetic Analyzer (Applied Biosystems). The insert was digested with EcoRI/Apa1 and subcloned into the same sites of the modified expression vector, pMXs‐AMNN‐puro. This vector was modified pMXs‐puro (kindly provided by T. Kitamura, University of Tokyo, Japan) by adding new restriction sites (ApaI, MluI, NruI, and NspV) to the multiple cloning site (MCS) of an empty vector in our laboratory.

### Stable revertant of Srebp‐1c

The Srebp‐1c revertant was generated using retroviral infection. The vectors, termed pMXs‐AMNN‐puro and pMXs‐AMNN‐puro‐Srebp‐1c mature form, were transfected into Plat‐E cells (kindly provided by T. Kitamura, University of Tokyo, Japan) with FuGENE^®^6 (Promega, Madison, WI, USA), according to the manufacturer's protocol. Each virus‐containing culture supernatant was collected 2 d after transfection and filtered through 0.22‐μm filters (Millipore, Billerica, MA, USA). To obtain stable cell lines, Srebp‐1c KO‐MEFs were incubated with virus‐containing medium for 2 days, followed by selection with 5 μg mL^−1^ puromycin for 5 days.

### Protein extraction and Western blotting

Western blotting was performed as described previously (Okita *et al*., 2012). The methods are briefly described as additional experimental procedures in Data S1 (Supporting information).

### Analysis of mitochondrial DNA (mtDNA) content and citrate synthase activity

The mtDNA content and CS activity were measured as previously described (Alp *et al*., 1976; Okita *et al*., 2012). The methods are briefly described as additional experimental procedures in Data S1 (Supporting information).

### Chromatin immunoprecipitation (ChIP) assay for WAT and MEFs

ChIP assays were performed as previously described with slight modifications. The methods are briefly described as additional experimental procedures in Data S1 (Supporting information).

### Analysis of oxidative stress

Total glutathione (GSH + GSSG) and GSSG levels were measured as previously described. Aconitase activity was measured with an Aconitase Assay Kit (Cayman Chemical, Ann Arbor, MI, USA) according to the manufacturer's protocol. The methods are described as additional experimental procedures in Data S1 (Supporting information).

### Statistical analysis

Data were represented as means ± SEM, and statistical significance was determined with Student's *t‐*test for comparing two groups or Tukey's test for comparing more than two groups after the assessment of significant differences by two‐ or three‐way analysis of variance (ANOVA). To compare survival distributions of AL and CR mice, the log‐rank test was applied. *P* values < 0.05 were considered statistically significant.

## Author contributions

Y.H. conceived the idea, designed experiments, and wrote the manuscript. N.F. and T.N. predominantly performed experiments, under the leadership of N.O., Y.S. and M.K. Y.F., Y.C., M.S., A.Y., and K.T. assisted in the experiments. I.S. supported the histological analysis. T.K. analyzed the data.

## Funding

This work was supported by Grants‐in‐Aid for Scientific Research (C) (No. 19590396) and for Challenging Exploratory Research (No. 26670193) from the Japan Society for the Promotion of Science and by the MEXT‐Supported Program for the Strategic Research Foundation at Private Universities, 2014–2018.

## Conflict of interest

We certify that there is no conflict of interest with any financial organization regarding the material discussed in the manuscript.

## Supporting information


**Fig. S1** Time‐course analysis of food consumption and body weights of mice.
**Fig. S2** Effects of CR on expression of proteins involved in FA biosynthesis in kidney, quadriceps femoris muscles (QFM) and heart tissues from mice.
**Fig. S3** Effects of CR on expression of proteins involved in the mitochondria in kidney, quadriceps femoris muscles (QFM) and heart tissues from mice.
**Fig. S4** Effects of CR on CS activity in various tissues from mice.
**Fig. S5** CR increased Srebp‐1 enrichment on the *Pgc‐1α* promoter in rat WAT.
**Fig. S6** Effects of CR on oxidative stress in various tissues from mice.
**Fig. S7** Analysis of survival curves.
**Table S1** Body and tissue weights when mice were euthanized at 8–10 months of age.
**Table S2** Raw data for survival of all mice.

**Data S1** Additional experimental procedures.
