# Supplementary material for: Sterol regulatory element‐binding protein‐1c orchestrates metabolic remodeling of white adipose tissue by caloric restriction
Source: Aging Cell. 2017 Mar 3;16(3):508–17. doi: 10.1111/acel.12576 (PMC5418191; doi:10.1111/acel.12576)
Supplement: Supplementary file 1 — Fig. S1 Time‐course analysis of food consumption and body weights of mice. Fig. S2 Effects of CR on expression of proteins involved in FA biosynthesis in kidney, quadriceps femoris muscles (QFM) and heart tissues from mice. Fig. S3 Effects of CR on expression of proteins involved in the mitochondria in kidney, quadriceps femoris muscles (QFM) and heart tissues from mice. Fig. S4 Effects of CR on CS activity in various tissues from mice. Fig. S5 CR increased Srebp‐1 enrichment on the Pgc‐1α promoter in rat WAT. Fig. S6 Effects of CR on oxidative stress in various tissues from mice. Fig. S7 Analysis of survival curves. Table S1 Body and tissue weights when mice were euthanized at 8–10 months of age. Table S2 Raw data for survival of all mice. [file ACEL-16-508-s001.pdf]

Figure S1

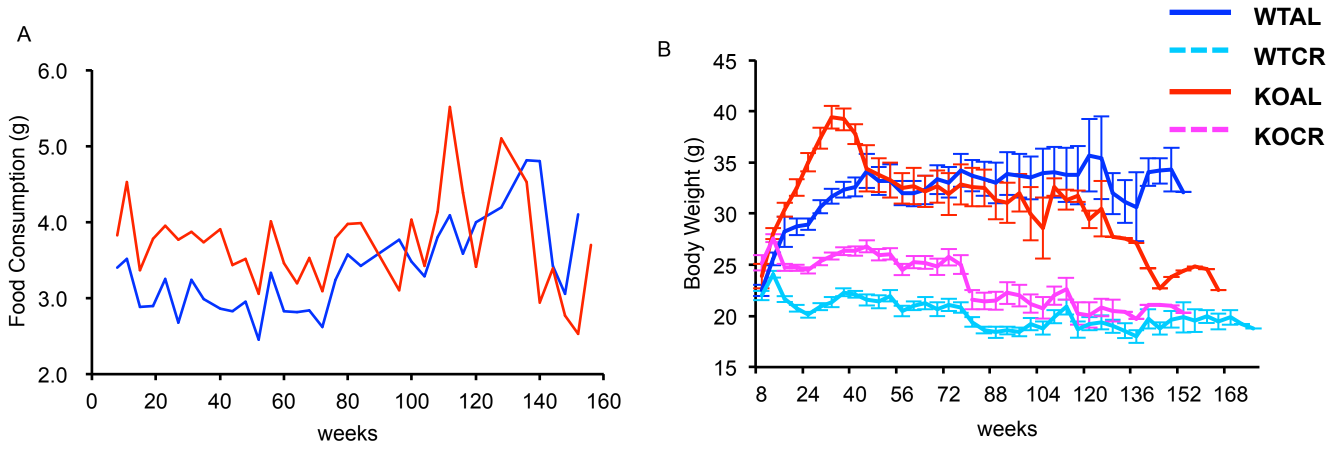

A
Food Consumption (g)
6.0
5.0
4.0
3.0
2.0
0
20
40
60
80
100
120
140
160
weeks
B
Body Weight (g)
45
40
35
30
25
20
15
8
24
40
56
72
88
104
120
136
152
168
weeks
WTAL
WTCR
KOAL
KOCR

Figure S2

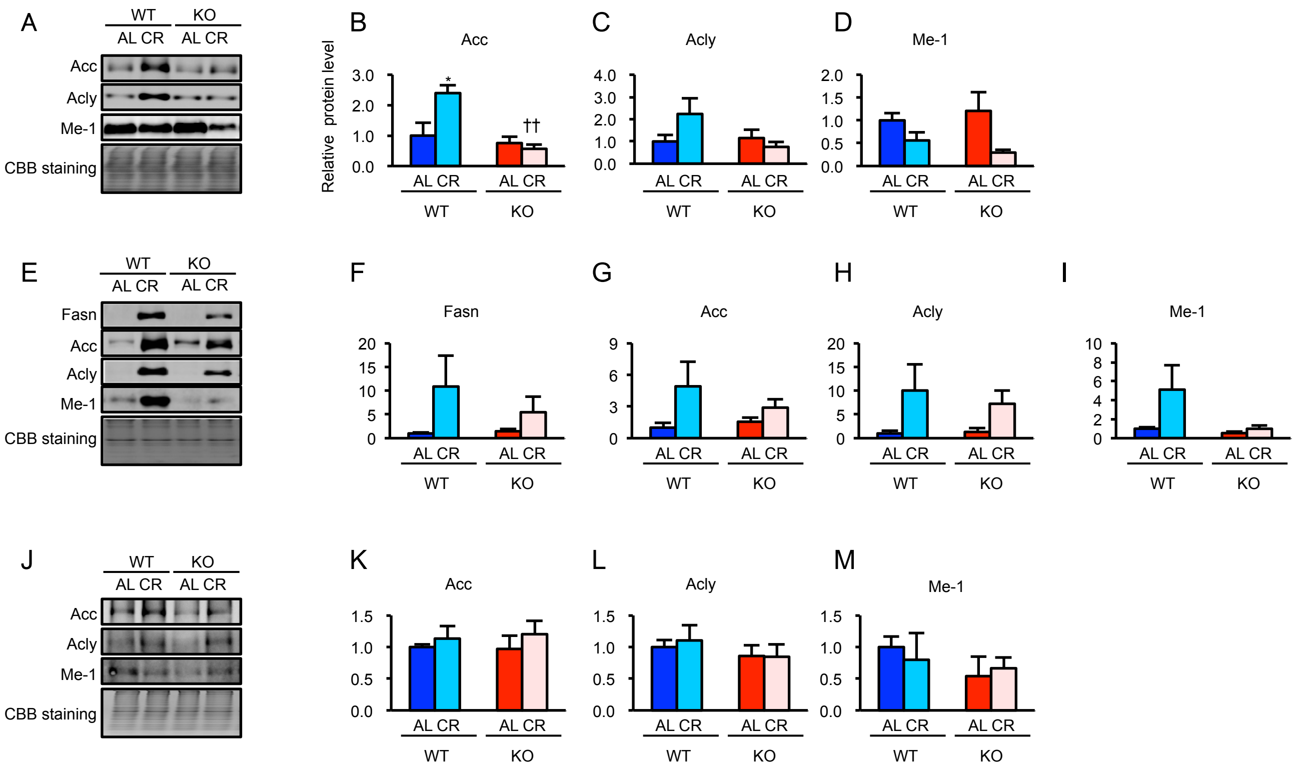

A
WT KO
AL CR AL CR
Acc
Acly
Me-1
CBB staining
B
Acc
Relative protein level
3.0
2.0
1.0
0.0
*
††
AL CR AL CR
WT KO
C
Acly
4.0
3.0
2.0
1.0
0.0
D
Me-1
2.0
1.5
1.0
0.5
0.0
E
Fasn
F
Fasn
20
15
10
5
0
G
Acc
9
6
3
H
Acly
I
Me-1
10
8
6
4
2
J
K
Acc
1.5
1.0
0.5
L
Acly
M
Me-1

Figure S3

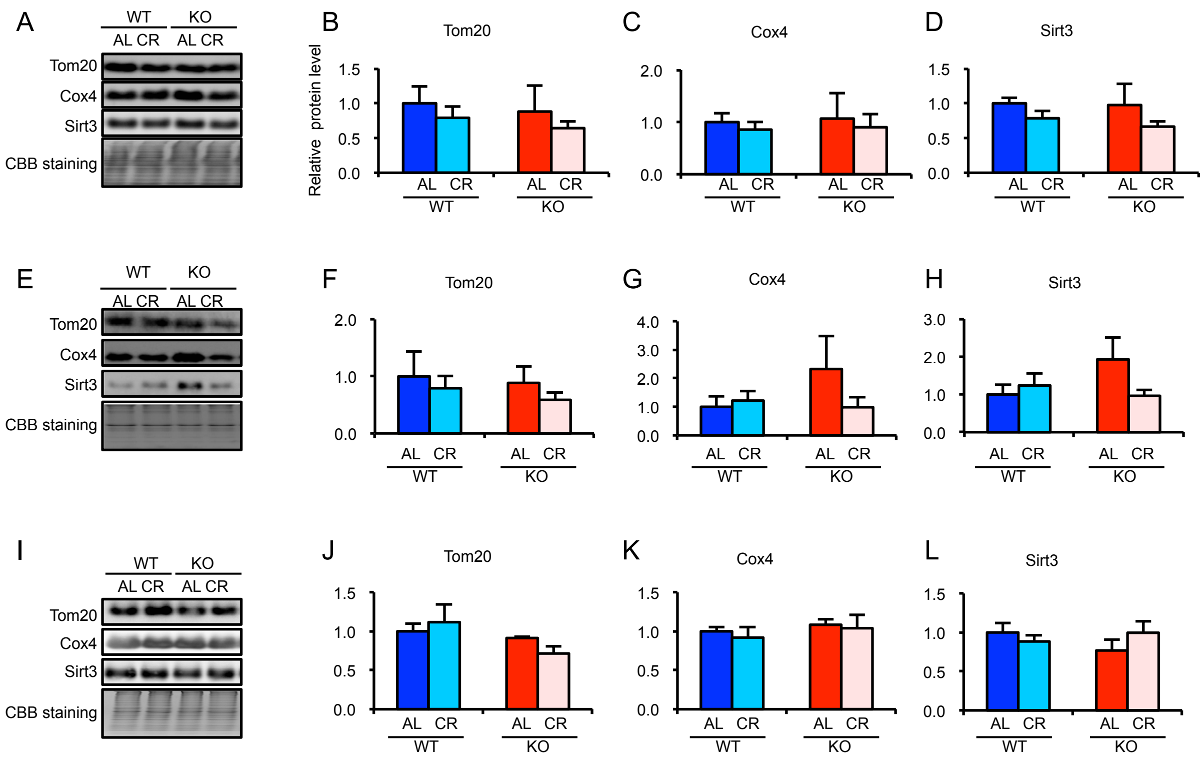

A
WT KO
AL CR AL CR
Tom20
Cox4
Sirt3
CBB staining
B
Tom20
Relative protein level
1.5
1.0
0.5
0.0
AL CR AL CR
WT KO
C
Cox4
2.0
1.0
0.0
AL CR AL CR
WT KO
D
Sirt3
1.5
1.0
0.5
0.0
AL CR AL CR
WT KO
E
WT KO
AL CR AL CR
Tom20
Cox4
Sirt3
CBB staining
F
Tom20
2.0
1.0
0.0
AL CR AL CR
WT KO
G
Cox4
4.0
3.0
2.0
1.0
0.0
AL CR AL CR
WT KO
H
Sirt3
3.0
2.0
1.0
0.0
AL CR AL CR
WT KO
I
WT KO
AL CR AL CR
Tom20
Cox4
Sirt3
CBB staining
J
Tom20
1.5
1.0
0.5
0.0
AL CR AL CR
WT KO
K
Cox4
1.5
1.0
0.5
0.0
AL CR AL CR
WT KO
L
Sirt3
1.5
1.0
0.5
0.0
AL CR AL CR
WT KO

Figure S4

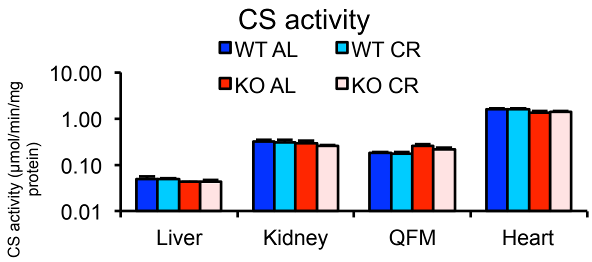
CS activity
WT AL
WT CR
KO AL
KO CR
CS activity (µmol/min/mg protein)
10.00
1.00
0.10
0.01
Liver
Kidney
QFM
Heart

Figure S5

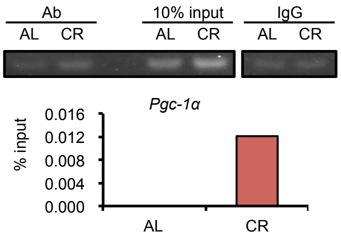
Ab
AL CR
10% input
AL CR
IgG
AL CR
Pgc-1α
% input
0.016
0.012
0.008
0.004
0.000
AL
CR

Figure S6

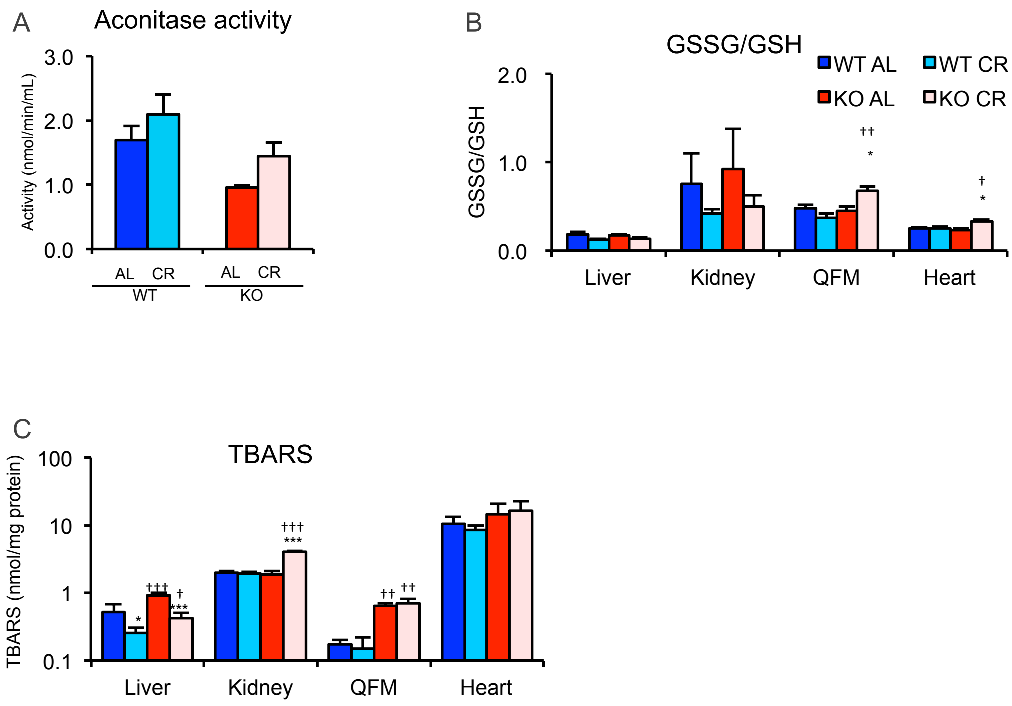
A
Aconitase activity
Activity (nmol/min/mL)
3.0
2.0
1.0
0.0
AL CR
WT
AL CR
KO
B
GSSG/GSH
WT AL
WT CR
KO AL
KO CR
GSSG/GSH
2.0
1.0
0.0
††
*
†
*
Liver
Kidney
QFM
Heart
C
TBARS
TBARS (nmol/mg protein)
100
10
1
0.1
†††
***
*
†††
†
***
††
††
Liver
Kidney
QFM
Heart

Figure S7

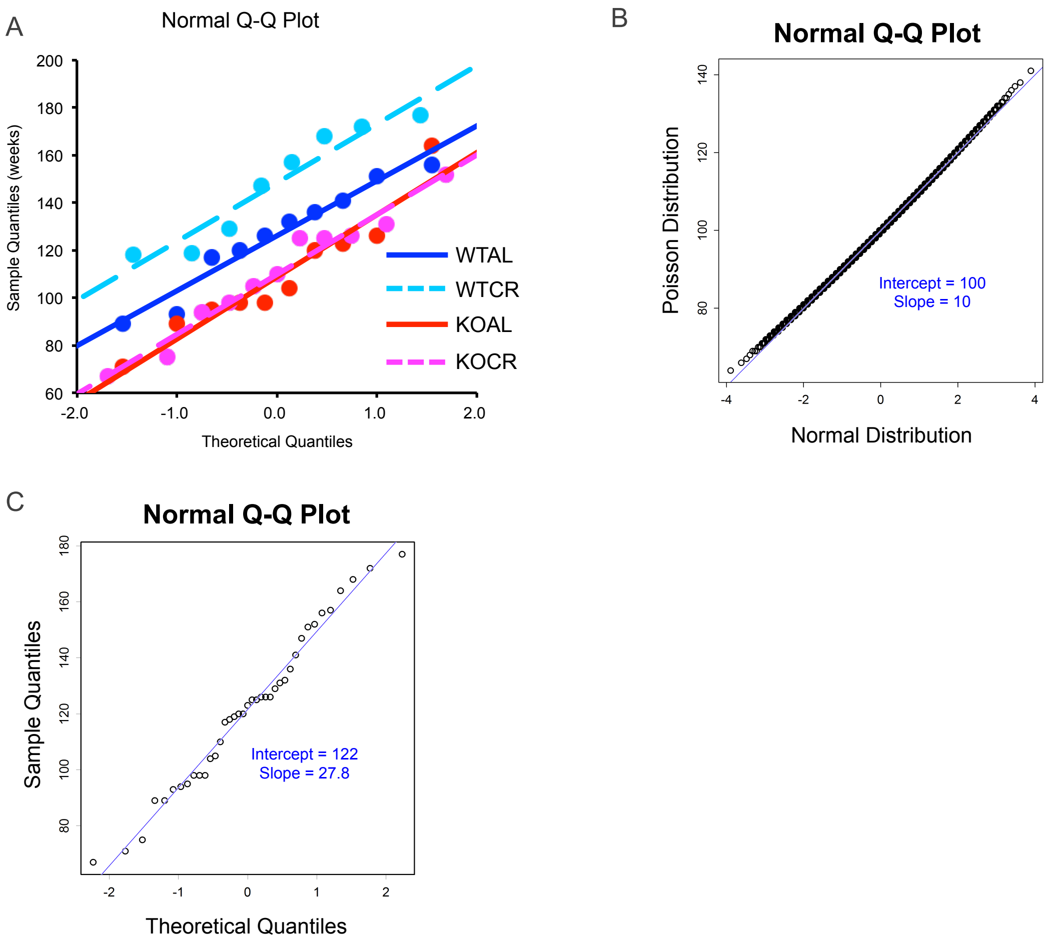
A
Normal Q-Q Plot
Sample Quantiles (weeks)
Theoretical Quantiles
WTAL
WTCR
KOAL
KOCR
B
Normal Q-Q Plot
Poisson Distribution
Normal Distribution
Intercept = 100
Slope = 10
C
Normal Q-Q Plot
Sample Quantiles
Theoretical Quantiles
Intercept = 122
Slope = 27.8

Table S1

Body and tissue weight		WT AL	WT CR		KO AL		KO CR	
Body weight	(g)	29.97 ± 0.89	21.78 ± 0.53	*	37.27 ± 1.81	†	26.11 ± 0.87	
Liver	(g)	1.018 ± 0.035	0.821 ± 0.024		1.358 ± 0.073	†	1.038 ± 0.037	*†
WAT	(g)	0.915 ± 0.105	0.230 ± 0.027	*	1.227 ± 0.152		0.327 ± 0.031	*
BAT	(g)	0.099 ± 0.008	0.078 ± 0.006		0.155 ± 0.025		0.086 ± 0.009	*
Heart	(g)	0.146 ± 0.004	0.119 ± 0.003		0.198 ± 0.010	†	0.150 ± 0.007	*†
Kidney	(g)	0.197 ± 0.006	0.144 ± 0.003	*	0.235 ± 0.012	†	0.156 ± 0.008	*

Each value represents the means ± S.E.M. *; $p<0.05$ vs. AL, †; $p<0.05$ vs WT analyzed by Tukey's test

Table S2

WTAL		WTCR		KOAL		KOCR	
Mouse	Age at Death (Weeks)	Mouse	Age at Death (Weeks)	Mouse	Age at Death (Weeks)	Mouse	Age at Death (Weeks)
1	89	1	118	1	71	1	67
2	93	2	119	2	89	2	75
3	117	3	129	3	95	3	94
4	120	4	147	4	98	4	98
5	126	5	157	5	104	5	105
6	132	6	168	6	120	6	110
7	136	7	172	7	123	7	125
8	141	8	177	8	126	8	125
9	151			9	152	9	126
10	156			10	164	10	131
						11	152
