# Supplementary material for: Sterol regulatory element‐binding protein‐1c orchestrates metabolic remodeling of white adipose tissue by caloric restriction
Source: Aging Cell. 2017 Mar 3;16(3):508–17. doi: 10.1111/acel.12576 (PMC5418191; doi:10.1111/acel.12576)
Supplement: Supplementary file 2 — Data S1 Additional experimental procedures. [file ACEL-16-508-s002.docx]

**Additional experimental procedures**

**Animals**

Male 5–7-week-old Wistar rats were purchased from Clea Inc. (Tokyo, Japan) and maintained under SPF conditions in the Laboratory Animal Center at the Faculty of Pharmaceutical Sciences, Tokyo University of Science. The animals, and their husbandry care and diet were previously described in detail (Okita et al., 2012). Briefly, from 12 weeks of age, the rats were divided into two groups: AL group and CR group (70% of *ad libitum* energy intake). At 9 months of age, rats were euthanized at 3–5 h after turning on the lights in the morning. CR rats were provided with food at 30 min prior to turning off the lights in the evening, and were euthanized the following morning. When the animals were euthanized, the epididymal WAT were collected and frozen in liquid nitrogen, stored at −80°C and used for ChIP assay.

**Quantitative real-time RT-PCR**

Total RNA was extracted from frozen WAT and liver using RNAiso PLUS (Takara, Japan), before purification with FastPure^TM^ RNA kit (Takara) according to manufacturer’s protocol (Chujo et al., 2013). To obtain cDNA, 1 μg of RNA was subjected to reverse transcription using PrimeScript^TM^ Reverse Transcriptase (Takara) with random hexamers (Takara). Quantitative real-time PCR (qRT-PCR) was performed using an Applied Biosystems 7300 real-time PCR system (Life Technologies, Carlsbad, CA) with SYBR^®^ Premix ExTaq^TM^II (Takara), according to manufacturer’s instructions (Chujo et al., 2013). Primer pair sequences for RT-PCR analysis included: *Adiponectin* (Forward: 5′-TGC CGA AGA TGA CGT TAC TAC AAC-3′, Reverse: 5′-CTT CAG CTC CTG TCA TTC CAA C-3′), *Cox4* (Forward: 5′-CAT TCC TAC TTC GGT GTG CCT TC-3′, Reverse: 5′-CAC ATC AGG CAA GGG GTA GTC-3′), *Fasn* (Forward: 5′- AGC AGG CAC ACA CAA TGG AC-3′, Reverse: 5′- GAA GAA GAA AGA GAG CCG GTT G-3′), *F4/80* (Forward: 5′- GGC CAA GAT TCT CTT CCT CAC-3′, Reverse: 5′- TCA CCA CCT TCA GGT TTC TCA C-3′), *γ-GCS* (Forward: 5′-CCA TCA TCA ATG GGA AGG AAG-3′, Reverse: 5′-TCC ACC TGG CAA CAG TCA TTA G-3′), *Mcp-1* (Forward: 5′- CCA GCC AAC TCT CAC TGA AGC-3′, Reverse: 5′- CTT CTT TGG GAC ACC TGC TG-3′), *Pgc-1α* (Forward: 5′-AGA GGG ATT GCC CTC ATT TG-3′, Reverse: 5′-CAG GGT TTG TTC TGA TCC TGT G-3′), *Pparg* (Forward: 5′-CAC AAT GCC ATC AGG TTT GG-3′, Reverse: 5′-GCG GGA AGG ACT TTA TGT ATG AG-3′), *Srebp-1a* (Forward: 5′-GGC CGA GAT GTG CGA ACT-3′, Reverse: 5′-TTG TTG ATG AGC TGG AGC ATG T-3′), *Srebp-1c* (Forward: 5′-GGA GCC ATG GAT TGC ACA TT-3′, Reverse: 5′-GGC CCG GGA AGT CAC TGT-3′), *Srebp-2* (Forward: 5′-GGA TCA AGT CAG CAG CCA AG-3′, Reverse: 5′-AAT CCC ACA GAG TCC ACA AAA G-3′) and *Tbp* (Forward: 5′-CAG TAC AGC AAT CAA CAT CTC AGC-3′, Reverse: 5′-CAA GTT TAC AGC CAA GAT TCA CG-3′). Amount of target mRNA was normalized to *Tbp* mRNA expression. In case of *Srebps* assay, to perform an absolute quantity, plasmids containing full-length cDNAs for *Srebp-1a*, *-1c*, or *-2*, encompassing the oligonucleotides used for PCR, were isolated and purified. DNA was quantified by A260 using a NanoDrop 1000 (Thermo Fisher Scientific, Waltham, MA). Serial dilutions of plasmid DNA were used as a standard curve for quantitation. Data from 4–5 mice in each group were expressed as mean ± SEM and examined using Tukey’s t test. Presence of significant statistical difference was inferred with p-values < 0.05.

**Protein extraction and western blotting**

Western blot was performed according to a previous report (Okita et al., 2012). Tissues and cells were lysed with lysis buffer (50 mM Tris-HCl, pH 6.8, 2% SDS and 5% glycerol), boiled for 5 min and sonicated. Protein concentrations within soluble fractions were determined using the Pierce BCA Protein Assay Kit (Thermo Scientific, Rockford, IL). The extracted protein samples were standardized by addition of lysis buffer, 2-mercaptoethanol and bromophenol blue to obtain final concentrations of 5% and 0.025%, respectively, before boiling samples for 5 min. Fifteen µg of each protein sample was subjected to SDS-PAGE and transferred to nitrocellulose membranes. Membranes were blocked with 2.5% skim milk and 0.25% BSA in Tris-buffered saline (50 mM Tris, pH 7.4, and 150 mM NaCl) containing 0.1% Tween 20 (TTBS) for 1 h at room temperature, and then probed with appropriate primary antibodies overnight at 4°C. Primary antibodies for Fasn (BD Biosciences, San Jose, CA), Acc (Cell Signaling, Boston, MA), Acly (Epitomics, Burlingame, CA), Me-1 (Sigma Aldrich), Tom20 (Sigma Aldrich), Cox4 (Cell Signaling), and Sirt3 (Cell Signaling) were used. After several washes with TTBS, membranes were incubated with an appropriate secondary antibody (horseradish peroxidase-conjugated F(ab')2 fragment of goat anti-mouse IgG or anti-rabbit IgG; Jackson ImmunoResearch, West Grove, PA) for 1 h at room temperature. After washing with TTBS, membranes were incubated with ImmunoStar^®^ LD reagent (Wako). Specific proteins were visualized with LAS3000 (Fujifilm, Japan), and data were analysed using Multigauge software (Fujifilm).

**Mitochondrial DNA (mtDNA) content**

mtDNA content was measured as previously reported (Okita *et al*., 2012). Briefly, total DNA was extracted and DNA and mtDNA were amplified by PCR for COX2 and β-Globin, respectively. Primer pair sequences for PCR included Cox2 (forward: 5′-CCA TCC CAG GCC GAC TAA-3′, reverse: 5′-AAT TTC AGA GCA TTG GCC ATA GA-3′) and β-Globin (Forward: 5′-ATC CAG GTT ACA AGG CAG CT-3′, Reverse: 5′-GGG AAA CAT AGA CAG GGG-3′). Relative amounts of mtDNA were expressed as COX2/β-Globin.

**Analysis of citrate synthase activity**

Activity of citrate synthase was measured as previously reported with some modification (Alp et al., 1976; Okita et al., 2012). Briefly, tissue was homogenized in buffer containing 50 mM Tris-HCl (pH 7.4), 150 mM NaCl, 1% phosphatase inhibitor cocktail (Thermo Scientific), 5 mM EDTA, 1% protease inhibitor cocktail (Sigma), 1% Triton X-100 and 0.05% sodium deoxycholate. Protein concentration was determined using a BCA protein assay kit according to the manufacturer’s protocol. For citrate synthase activity measurements, a reaction mixture containing 0.1 mM 5,5-dithio-bis-(2-nitrobenzoic) acid (Wako), 0.5 mM acetyl-CoA (Wako), 0.1% Triton X-100 and 100 mM Tris-HCl (pH 8.0) was added to tissue homogenate. Homogenates contained 5–8 µg of protein from tissue. After incubation at 28 ºC for 5 min, absorbance at 412 nm (SpectraMax Plus384, Molecular Devices) was measured for 3 min to determine nonspecific activity. Reactions were then initiated by addition of 0.5 mM oxaloacetate (Wako) in a final volume of 200 µL, with change in absorbance recorded for at least 3 min.

**Chromatin immunoprecipitation (ChIP) assay for white adipose tissue and MEFs**.

ChIP assays were performed using previously reported methods with slight modification (Kotake et al., 2007; Kuo and Allis, 1999). For WAT ChIP assay, 400 mg of WAT was lysed in cold PBS containing 0.3% Nonidet P-40 (NP-40) and protease inhibitor cocktail. WAT was fixed with 1% formaldehyde for 10 min at room temperature. Cross-linking was stopped by addition of glycine to a 480 mM final concentration. WAT was homogenized, washed with cold PBS, and pelleted by centrifugation. MEFs were grown to 50–60% confluency and then fixed with 1% formaldehyde for 10 min at room temperature. After washing with PBS, cross-linking reaction was stopped by addition of glycine to 125 mM final concentration. Subsequently, cells were washed with cold PBS, harvested in PBS containing protease inhibitors cocktail, and pelleted by centrifugation.

WAT-derived cross-linked pellets and cross-linked cell pellets were resuspended in cell lysis buffer (10 mM HEPES-KOH (pH 7.9), 1.5 mM MgCl_2_, 10 mM KCl, 0.5 mM dithiothreitol and 0.5% NP-40) and pelleted. Nuclei were lysed in SDS lysis buffer (50mM Tris-HCl (pH 8.0), 10 mM EDTA and 1% SDS) and samples were sonicated to generate DNA fragments of less than 500 bp.

For immunoprecipitation, approximately 200 µg of protein extracts were incubated overnight with 4 µg anti-Srebp-1 antibody (Santa Cruz Biotechnology) or nonspecific mouse immunoglobulin G. Next, 30 µL of 50% protein G agarose slurry (precleared for 2 min, 3 times) were added to antibody-antigen mixtures and incubated for 1 hr. Beads were washed twice with low salt buffer (20 mM Tris-HCl (pH 8.0), 150 mM NaCl, 2.0 mM EDTA, 1.0% Triton-X 100 and 0.1% SDS), twice with high salt buffer (20 mM Tris-HCl (pH 8.0), 500 mM NaCl, 2 mM EDTA, 1.0% Triton-X 100 and 0.1% SDS), twice with LiCl Buffer (10 mM Tris-HCl (pH 8.0), 250 mM LiCl, 1 mM EDTA, 1.0% NP-40 and 1.0% deoxycholic acid), and four times with 1X TE (10 mM Tris-HCl (pH 8.0) and 0.1 mM EDTA). Cross-linking was reversed by incubating samples overnight at 65°C with ChIP elution buffer (For WAT: 0.1 M NaHCO_3_, 0.2 mM NaCl, 10 mM dithiothreitol, 1.0% SDS; and for MEFs: 10 mM Tris-HCl (pH 8.0), 300 mM NaCl, 5 mM EDTA, 0.5% SDS). DNA was purified and a fraction was used as template in PCR reactions. PCR reactions were performed using KOD FX neo (Toyobo, Japan). Primer pair sequences used were previously reported: Fasn (Forward: 5′-CCA GTG TGA CCA AGC ACG CC-3′, Reverse: 5′-GCG CTG GAG CAC AAG GAA CG-3′) (Gosmain et al., 2005), Pgc-1α (Forward: 5′-AGT GAC AGC CCA GCC TAC TTT-3′, Reverse: 5′-AGC CCC TTA CTG AGA GTG AAC-3′) and β-globin (Forward: 5′-CCT GCC CTC TCT ATC CTG TG-3′, Reverse: 5′-GCA AAT GTG TTG CCA AAA AG-3′) (Hao et al., 2010). Input DNA values were used to normalize values obtained from ChIP samples.

**Analysis of oxidative stress**

Total glutathione (GSH + GSSG) and GSSG levels were measured by a previously reported method (Rahman et al., 2006). Briefly, WAT, QFM and heart were homogenized in extraction buffer (0.1 M potassium phosphate buffer with 5 mM EDTA (pH7.5), 0.1% Triton X-100 and 0.6% sulfosalicylic acid) and centrifuged at 4 ºC for 10 min. Liver and kidney were homogenized with 5% metaphosphoric acid in extraction buffer, then centrifuged at 4 ºC for 10 min. Supernatants were used for measurement of tissue glutathione contents. Rates of 2-nitro-5-thiobenzoic acid (DTNB) formation were calculated and total glutathione (tGSH) and GSSG concentrations present in samples were determined using linear regression to calculate values obtained from a standard curve. GSH concentration was calculated by subtracting GSSG concentration from the tGSH concentration.

Activity of aconitase was measured with an Aconitase Assay Kit (Cayman Chemical, Ann Arbor, MI) according to manufacturer’s protocol.

Thiobarbituric acid reactive substances were measured by a previously reported method with slight modification (Ohkawa et al., 1979). Liver, heart, QFM and kidney tissues were homogenized in 154 mM KCl (1:10 w/v). A reaction mixture containing 0.477% SDS, 8.82% acetic acid and 0.353% thiobarbituric acid was added to tissue homogenate and incubated for 30 min on ice. Next, samples were incubated at 95ºC for 1 hr, before adding equal butanol/pyridine (15:1, v/v) and centrifuging at 25ºC for 10 min. Absorbance of supernatant was measured at 532 nm using a SpectraMax Plus 384 (Molecular Devices). For standards, serial dilutions of malondialdehyde (Wako) were used.

**Distribution of longevity could be approximated by the normal distribution**

If a mouse die spontaneously under certain probability, its potential longevity will obey Poisson distribution, which is defined by a single parameter λ, which is determined by the probability. Even among mice under group feeding, the probability may vary, and it may be altered upon time. Then, distribution of longevities in a group of mice would be mixture of Poisson distributions that vary in λ.

When λ is large enough, Poisson distribution could be approximated by using the normal distribution (Fig. S7B). Therefore, it is possible that mixture of Poisson distributions could be approximated with single normal distribution, if the differences among λ parameters are not too large. The scale parameter of the normal distribution, σ, will become larger than expected from the location parameter μ as μ2 = σ, because the differences among λ will widen the distribution, enlarging σ.

As is expected, normal distribution well approximated that of longevities obtained in the whole experiment (Fig. S7C); the slope and intercept of the regression present σ and μ of the approximating distribution, respectively. The σ parameter was more than twice of square root of μ, showing that σ had been determined by the differences of λ parameters, rather than the magnitude of μ. The normal character was also observed in each group of data (Fig. 6H in the text).

The approximation enables to apply a parametric model to testing significance of longevity differences. In a strict sense, the population is not really normal but mixture of several distributions that have smaller scales, so estimated scale would become larger than the real magnitude of noise. Noise for each sample would be expected as square root of λ, but that estimated by the experiment would be the slope for the mixture (Fig. S7C). This would make the test conservative.

Regression lines were estimated by a robust method, using the lm() function of the R. Whether extension of longevity by calorie restriction was reproduced among the groups was parametrically tested, using Student’s t-test. As we can expect that calorie restriction enlarges longevity (Chung et al. 2013), whether it is reproduced in the presented experiment or not was tested. Hence one-tailed test assuming an equal variance was applied.

**References**

Kotake Y, Cao R, Viatour P, Sage J, Zhang Y, Xiong Y (2007) pRB family proteins are required for H3K27 trimethylation and polycomb repression complexes binding to and silencing p16INK4alpha tumor suppressor gene. *Genes Dev.* **21**, 49-54.

Kuo MH, Allis CD (1999) In vivo cross-linking and immunoprecipitation for studying dynamic protein:DNA associations in a chromatin environment. *Method* **19**, 425-433.

Rahman I, Kode A, Biswas SK (2006) Assay for quantitative determination of glutathione and glutathione disulfide levels using enzymatic recycling method. *Nat. Protoc.* **1**, 3159-3165.
