# Supplementary material for: Sterol regulatory element‐binding protein‐1c orchestrates metabolic remodeling of white adipose tissue by caloric restriction
Source: Aging Cell. 2017 Mar 3;16(3):508–17. doi: 10.1111/acel.12576 (PMC5418191; doi:10.1111/acel.12576)
Supplement: Supplementary file 3 [file ACEL-16-508-s003.docx]

Table S1. Body weight and tissue weight of 4 groups of mice under fasted condition when mice were euthanized at 8–10 months of age. Values shown in all panels are means ± SEM. *: p<0.05 vs AL, †: p<0.05 vs. WT analyzed by Tukey’s t test.

Table S2. Raw data for survival of all mice.

Fig. S1 Time-course analysis of food consumption and body weight. Food consumption (A) and body weight (B) were measured every four weeks. Values shown in two panels are means ± SEM.

Fig. S2 Effects of CR on expression of proteins involved in FA biosynthesis and mitochondria in kidney, quadriceps femoris muscles (QFM) and heart of WT and KO. (A–D) Kidney, (E–I) QFM and (J–M) heart. Example of immunoblot images showing expression of proteins involved in FA biosynthesis in kidney (A), QFM (E) and (J) from four groups of fed mice (n=3–5 per group). Quantitative analysis was performed using chemiluminescence method. Results of Fasn (F), Acc (B, G, K), Acly (C, H, L) and Me-1 (D, I, M) are expressed as relative intensity of indicated protein/CBB staining compared with WTAL. Values shown in all panels are means ± SEM with analysis by Tukey’s t test.

Fig. S3 Effects of CR on expression of proteins involved in mitochondria in kidney, quadriceps femoris muscles (QFM) and heart of WT and KO. (A–D) Kidney, (E–H) QFM and (I–L) heart. Example of immunoblot images showing expression of proteins involved in mitochondria in kidney (A), QFM (E) and heart (I) from four groups of fed mice (n=3–5 per group). Quantitative analysis was performed using chemiluminescence method. Results of Tom20 (B, F, J), Cox4 (C, G, K) and Sirt3 (D, H, L) are expressed as relative intensity of indicated protein/CBB staining compared with WTAL. Values shown in all panels are means ± SEM with analysis by Tukey’s t test.

Fig. S4 (A) CS activity in liver, heart, QFM and kidney from four groups of fed mice was measured spectrophotometrically at 412 nm. Values shown in the panel are means ± SEM with analysis by Tukey’s t test.

Fig. S5 (A) CR increases Srebp-1 enrichment on *Pgc-1α* promoter in rat WAT. Representative gel image of chromatin immunoprecipitation (ChIP) assay for Srebp-1c enrichment on *Pgc-1α* promoter. PCRs for promoter of *Pgc-1α* genes were performed using the DNA pulled down with the Srebp-1 antibody-transcription factor complexes in WAT. % Input was calculated using the formula: 2^(Ct [1% of input]−Ct [ChIP])^ . For ChIP assays, IgG was used as negative control. Experiments were duplicated.

Fig. S6 Effects of CR on oxidative stress in various tissues of WT and KO. Various biomarkers for oxidative stress were measured in several tissues from four groups of fed mice (n=3–6 per group). (A) Aconitase activity of liver was spectrophotometrically measured by NADPH formation at 340 nm. (B) oxidized glutathione (GSSG) to glutathione (GSH) ratio was measured spectrophotometrically at 412 nm by DTNB-GSSG reductase recycling assay. (C) Thiobarbituric acid reactive substance (TBARS) level, as a lipid peroxidation marker, was measured spectrophotometrically at 532 nm and normalized by protein content. Values shown in all panels are means ± SEM. *: p<0.05, ***: p<0.001 vs AL, †: p<0.05, ††: p<0.01, †††: p<0.001 vs. WT analyzed by Tukey’s t test.

Fig. S7 (A) Survival analysis. 8–11 mice in four groups were maintained under pathogen-free conditions. Normal QQ plot of the longevities for each group are shown. Linear relationships indicate the normality of data distribution. Numbers in the legend show the intercepts and slopes of the regression lines. (B) Comparison between quantiles of the normalization and Poisson distribution (λ = 100). Regression line was drawn by using the normal distribution (N:0,1) with the indicated parameters: square root of the intercept coincided with the slope. (C) Normal Quantile-quantile plot for longevity of mice studied. A straight relationship indicates normality of the distribution.
